# Applicability and usefulness of the Declaration of Helsinki for forensic research with human cadavers and remains

**DOI:** 10.1007/s12024-022-00510-4

**Published:** 2022-08-06

**Authors:** Valentina Scarpulla, Alberto Amadasi, Susi Pelotti, Francesca Ingravallo

**Affiliations:** 1 Department of Medical and Surgical Sciences (DIMEC), Alma Mater Studiorum, University of Bologna, Via Irnerio 49, 40126 Bologna, Italy; 2 Institute of Legal Medicine and Forensic Sciences, University Medical Centre Charité, University of Berlin, Berlin, Germany

**Keywords:** Forensic medicine, Legal medicine, Forensic anthropology, Cadaver, Ethics, Risk assessment

## Abstract

Bodies of deceased persons and human remains and their specimens (i.e., organs, bones, tissues, or biological samples) are essential in forensic research but ad hoc worldwide-recognized ethical standards for their use are still lacking. Such standards are needed both to avoid possible unethical practices and to sustain research in the forensic field. Pending consensus within the forensic science community regarding this topic, with this article we aim to stimulate a debate as to the applicability and usefulness of the Declaration of Helsinki in the field of forensic research involving human cadavers and remains. Considering the fundamental differences compared to clinical research involving human beings and the different moral obligations involved, we focus on the risks, burdens, and benefits of research, ethics committee approval, and informed consent requirements. The Declaration of Helsinki framework allows forensic researchers to focus on substantial ethical principles promoting the consistency, integrity, and quality of research. Consensus regarding ethical standards and the adoption of national and supranational laws that clearly regulate the use of human cadavers and remains, including those from autopsies, continues to be of primary importance for the forensic science community.

## Introduction

Forensic research applies several methods to many different types of research that may involve human subjects, human material obtained from either living persons or dead persons, including the whole body, and personal data. In particular, the use of human cadavers and remains (hereinafter HC&R), or their specimens (i.e., organs, bones, tissues, or biological samples), is essential for advancement in forensic research [1] but may raise ethical concerns [1–7]. Indeed, as suggested by Jones and Whitaker [8] “the manner in which we respond to the dead, the use we make of their skeletal remains and their tissues, and the ways in which we learn about ourselves by studying them, raises ethical queries that go to the heart of what it means to be human.” Accordingly, forensic doctors and assistants have reported that the dignity of the body is a central issue in everyday forensic professional practice [9].

However, while research on living people is subject to intense ethical scrutiny today [10], and ethics guidelines have been proposed for anatomical dissection [11] or for research with recently deceased and brain dead cadavers [12], no ethical standards or guidelines for forensic research involving HC&R are available internationally. This may be, at least in part, the result of a lack of consensus among forensic pathologists regarding the ethical acceptability of diverse uses of autopsy tissues for research [2]. On the other hand, ethical standards are needed both to avoid possible unethical practices and to sustain research in the forensic field. Indeed, in the absence of guidelines, ethical issues such as confidentiality and the use of “non-consented autopsy tissues” were among the main limitations to research reported by US academic forensic pathologists [13].

A cornerstone of biomedical research involving human subjects is the Declaration of Helsinki issued by the World Medical Association (WMA) [14]. The Declaration of Helsinki, the first version of which dates to 1964, was not issued in order to be legally binding, but many national laws, and the *Regulation on clinical trials on medicinal products for human use* recently adopted by the European Parliament and the Council [15], require its principles to be followed in all medical research involving human beings.

According to the Preamble, the WMA developed the Declaration of Helsinki “as a statement of ethical principles for medical research involving human subjects, including research on identifiable human material and data,” and “encourages others [than physicians] who are involved in medical research involving human subjects to adopt these principles” [14]. These premises fit very well with forensic research that is characterized by both the use of human material and data and the involvement of researchers with non-medical backgrounds.

However, deceased individuals are not considered human subjects in some countries, as is the case under US federal regulations, or HC&R may be deemed as *res nullius*, with little oversight, consequently, of their use [16, 17]. In addition, while almost all the Declaration of Helsinki principles are applicable in forensic research with HC&R, with only a few statements not applicable (e.g., those related to the use of placebo or post-trial provisions), the vast differences between clinical research involving human beings and forensic research involving HC&R stand out.

Bearing these aspects in mind, in the following paragraphs we discuss the main problems and benefits of the applicability of the Declaration of Helsinki to forensic research on HC&R, especially in relation to the provisions concerning risks, burdens, and benefits involved with research, ethics committee approval, and informed consent requirements. Our aim is to stimulate discussion within the forensic scientific community regarding this topic. Since laws vary from country to country, we will consider the Declaration of Helsinki in terms of its global value and we will mention national and supranational laws or regulations when needed in order to better contextualize the issues concerning the use of HC&R for forensic research purposes.

## Risks, burdens, and benefits of forensic research

Protecting people from the risk of being harmed because of their involvement in research is the main concern of research ethics [18]. The Declaration of Helsinki provides clear guidance regarding the need for careful assessment of predictable risks, burdens, and benefits, the implementation of measures to minimize the risk, and continuous monitoring, assessment, and documentation of risks.

Of course, in the case of forensic research involving HC&R, the risks and burdens are different from those that may harm a living person. On the other hand, as summarized by Pentz and colleagues, “even though they are not identical, there is direct continuity between the body of a person who has died and the living person” [12]. Therefore, when handling HC&R, particular attention should be paid to those behaviors that may undermine the dignity of the individual to whom the sample belonged. Which ethical limits should not be exceeded in the use of HC&R for forensic research purposes? The answer to this question encompasses scientific, ethical, cultural, religious, and psychological aspects. Moreover, professionals dealing with HC&R should develop procedures to ensure that donor programs and the use of cadaveric material reflect progress in ethical awareness within a geographical context [19].

When dealing with human cadavers, the first focus is not on the body itself, but upon the person to whom the body and the remains belonged ante-mortem and belong post-mortem [20]. The cadaver should be respected as a symbol of the living person [21], and should be handled in a dignified manner [9]. It was suggested that human remains deserve to be treated with the same respect and dignity [6, 22].

In addition, it should be considered that some individuals or groups may accord a “special status” to the body and its parts [23], due to different religious beliefs [24, 25] or thoughts [26]. It is, therefore, of paramount importance to assess the ethics of research involving HC&R “with an awareness of and sensitivity to the known values, beliefs and attitudes of those from whom the materials originated” [23]. With specific reference to these aspects, the research protocol should provide not only the scientific background of the research, but also clear information about how to carry it out with regard to the collection, use, storage, and destiny of HC&R.

Research lacking sufficient scientific background, or characterized by unnecessary loss or damage of samples, should not be considered to be ethically acceptable. For instance, according to Hostiuc [4], the use of whole cadavers to study the biomechanics of falls from various heights cannot be considered acceptable considering the existence of alternative methods (e.g., molds). With regard to specimens, careful handling is crucial when they are taken, brought to the laboratory, stored, analyzed, and eventually destroyed.

Research involving HC&R may also imply several risks with regard to data protection, especially in the case of identifiability of the body or specimens [17], and when genetic testing is involved [7]. Therefore, the research protocol should address risks for the deceased individual’s privacy, detailing how confidentiality will be assured, as well as risks for family members or ethnic groups in the case of genetic testing. Policies to manage incidental findings [27] should be included if appropriate.

Remarkably, in forensic research involving HC&R, risks and burdens could concern researchers themselves, as became evident in the case of COVID-19 forensic autopsies performed for scientific purposes [28, 29]. As suggested by Sperhake [28], “every corpse must be considered potentially infectious.” Indeed, biological risks may be present both when carrying out autopsies and handling human remains through materials or soil (e.g., tetanus or diphtheria) [30, 31]. Therefore, the protection of researchers’ health is an issue that also ought to be thoroughly considered: safety in the workplace should be guaranteed, research methods should be evaluated critically in advance and monitored, and individual protective equipment must always be available and used. Dead bodies do not pose serious risks to the public generally and for those handling them if the morgue attendants and pathologists follow recommended biocontainment precautions in autopsy practice and in the transportation of human remains. Safety in managing HC&R should therefore be considered itself an ethical priority both to protect those involved in the research and to avoid misconceptions about body infectivity and unnecessary public worries with regard to HC&R. The biological risk related to HC&R should not be overestimated, and may and should be managed according to existing safety recommendations.

Finally, the overall implications of the research should be predicted in terms of potential benefits and application. In many medical disciplines, body donor programs have already provided useful contributions to medical research. The availability of human body parts is of the utmost importance for basic research and for clinical research aimed at improving therapies or developing new treatments. The benefits of forensic research do not only concern advancements in knowledge within the forensic field, such as the improvements made in the assessment of the cause of death by virtopsy [32]. Indeed, even if burdened by an apparently non-therapeutic interest, the value of forensic research for third parties and the entire community has long been established [33]. The advantages of forensic research involving HC&R may include benefits for family members when the research results allow them to adopt preventive strategies, and benefits for society, when the results may help to prevent or treat diseases or avoid accidents. Indeed, according to Byard [34], preventive pathology extends beyond accidents at any age, also addressing suicides, homicides, and certain heritable and non-heritable diseases. As recently outlined with regard to the COVID-19 pandemic, “the scientific benefit that can be drawn from experience with autopsies and further examination of tissue samples is immeasurable” [28]. With this regard, early in the pandemic, the forensic community contributed to bring new knowledge to the pathophysiology and immunopathology of severe COVID-19 [35].

The prevalence of benefits over risks is one of the main arguments supporting forensic research in the sensitive field of forensic taphonomy. The first human taphonomy research center was created in 1981 at the University of Tennessee (Knoxville, USA), and currently there are several centers in the USA, one in Australia, and one in The Netherlands [36]. These facilities provide “a unique opportunity in the forensic sciences to study human decomposition using cadavers in a controlled research environment” [37]. Indeed, animal proxies do not seem to provide sufficiently accurate data regarding the time of death for a human being [36, 37]. The ethical issues of the use of HC&R in these human decomposition facilities have been extensively addressed, and guidelines and best practices have been proposed [36, 38].

## Research ethics committee approval and protocol registration

A second major issue of interest concerns the ethical scrutiny of research. While authorization of an ethics committee is usually needed before starting any research on both animal and human subjects worldwide, similar authorization for the use of HC&R for scientific purposes is only mandatory, in specific circumstances, in a few countries. For instance, a protocol approval by an ethics committee is required for body donation programs in Italy [39], it is also required in Spain before the removal of body material after death when the subject’s wishes are unknown and are impossible to discover [40], and it is necessary for research projects involving human remains in Norway [41].

On the other hand, according to the Declaration of Helsinki, every protocol “must be submitted for consideration, comment, guidance and approval to the concerned research ethics committee before the study begins” (art. 23) [14]. Even if this provision may be time-consuming and sometimes challenging for forensic researchers, the importance of a formal institutional approval for research projects involving the use of HC&R has been consistently underlined [3, 6, 42], and has several advantages for forensic research.

First, the scrutiny of a research ethics committee helps forensic researchers to align the study protocol with the complex regulations concerning data protection and informed consent established in each country. Auditing by an ethics committee may be fundamental in order to establish whether or not the informed consent process and forms are required and appropriate, and whether broad consent is acceptable in the specific case in question [4]. Second, ethics committee approval may potentially increase the numbers of body donations for forensic research. Indeed, fear or anxiety regarding possible disrespectful behavior towards cadavers was consistently found to be a reason for not donating bodies over time [43–45]. These concerns may be in part related to media cases regarding unauthorized storage and use of human samples, such as the instance of the Bristol Royal Infirmary and of the Liverpool Alder Hey Children’s Hospital [46]. Scrutiny by the research ethics committee prevents ethically unacceptable studies from being carried out, and represents a guarantee of ethical integrity of the research for both the donor and family members. Finally, a systematic submission of forensic research protocols to the ethics committee for approval may prevent regrettable situations for both biomedical journal editors requiring information on this issue and researchers dealing with the publication of study results.

A closely-related key point is the registration protocol in publicly accessible databases. To the best of our knowledge, no study has addressed this issue yet with regard to forensic research involving the use of HC&R. However, trial registration could be fundamental for forensic research for many reasons: the unintended duplication of existing studies could be avoided, results from different studies could be compared, thus expanding scientific knowledge, and the publication of selective results could be prevented. In addition, protocol registration could increase body donations thanks to the publication of the purpose of the research, encouraging the interest of possible donors.

An example of a public database is ClinicalTrials.gov (https://clinicaltrials.gov/), which is a registration and results database for clinical studies conducted around the world. At the present time, this register contains only a few forensic protocols involving HC&R. Since a public register for forensic research is lacking, the registration of forensic study protocols in one of the existing clinical trial public registers should be encouraged.

## Informed consent issues

The sensitive issue of informed consent to forensic research on HC&R has been addressed by several scholars [1, 4–6] to the work of whom we refer the readers. An in-depth examination of this issue, including a list of the various cases, goes beyond the objectives of this article.

In summary, apart from very few exceptions [3], research should only be carried out with the free and informed consent of a competent person. This means that a competent person, after having been adequately informed as to the potential use of the body and its parts, would donate his/her body or biological samples for forensic research purposes. Indeed, only through detailed information can the donors freely choose whether or not to give their consent [47]. However, as shown by Bach [17], scarce ethical guidelines and regulatory oversight have allowed worrying practices with regard to body donation for research: people are often poorly informed or misled as to the risks, and some tissue banks use language that may even be potentially exploitative in their advertisements.

In this regard, the Declaration of Helsinki is a very useful reference when developing informative material for donation programs. Indeed, according to the Declaration of Helsinki, “each potential subject must be informed adequately of the aims, methods, sources of funding, any possible conflicts of interest, institutional affiliation of the researcher, the anticipated benefits and potential risks of the study and the discomfort it may entail, post-study provisions and any other relevant aspects of the study” (art. 26) [14]. Applied to forensic research, this means that potential donors and their next of kin should at least be provided with the following information: for what purposes the body can be used, what invasive—to a greater or lesser extent—interventions or destructive actions may be performed (autopsy, sampling of organs, post-mortem biopsies, various research methods, recovery of bones, etc.), how many years the sample can be held in storage by the department, and what fate may be chosen at the end of the retention time (cremation, burial, return to family, etc.). After verifying that all the information has been clearly understood, written informed consent should be acquired for both the body donation and the collection and storage of personal data by the department, and for the use of the results provided by the scientific research. The potential subject should be informed of the right to withdraw consent at any time and with regard to communication of incidental findings to family members.

However, body donation for research purposes is neither a common nor an accepted practice worldwide [48], and in the daily practice of forensic research body donation is even less common. While compliance with the deceased person’s wishes, goals, and values is one of the requirements for a respectful use of HC&R, in the forensic field it is not easy to obtain informed consent before death, which is, in most cases, unexpected. In some situations, not only the wishes but even the very identity of the deceased may be, and may remain, unknown. In these instances, regulations such as the UK Human Tissue Act [49] or the EU Recommendation CM/Rec(2016)6 on research on biological materials of human origin [50] are of little help. Furthermore, the use of biological materials obtained during forensic autopsies is poorly or inadequately regulated by the law in most countries [3–5].

On the other hand, forensic researchers could rely on article 32 of the Declaration of Helsinki concerning “medical research using identifiable human material or data, such as research on material or data contained in biobanks or similar repositories,” that establishes that in “exceptional situations where consent would be impossible or impracticable to obtain for such research […] the research may be done only after consideration and approval of a research ethics committee” [14]. We argue that this statement could also be applied to the use of both unclaimed bodies, which still represent an important source for forensic research, and of human remains from unknown people.

## Conclusions

The absence of ad hoc ethical guidelines for forensic research involving HC&R may represent a barrier for research and expose researchers to the risk of ethically questionable practices, especially in countries where this issue is poorly or inconsistently regulated by the law. Bearing in mind the inherent differences in comparison with clinical research involving human beings and the different moral obligations involved, the Declaration of Helsinki is applicable and useful for forensic research involving HC&R. Indeed, the Declaration of Helsinki framework allows researchers to focus on substantial ethical principles and issues, promoting the consistency, integrity, and quality of forensic research.

Of course, the Declaration of Helsinki is not a panacea for forensic research. Consensus regarding ethical standards and the adoption of national and supranational laws that clearly regulate the use of human cadavers and remains, including those from autopsies, continued to be of primary importance for the forensic science community. However, the systematic and publicized adoption of the Declaration of Helsinki principles, along with improved visibility and transparency of forensic research through the registration protocol in public databases, could increase public trust in forensic research and, ultimately, increase “good” research in the field. Editors of forensic science journals could play a pivotal role, by uniformly adopting publication policies that set the Declaration of Helsinki as the ethical standard for research involving human biological material.

## Key points

1. Bodies of deceased persons and human remains are essential in forensic research but ad hoc worldwide-recognized ethical standards for their use are still lacking.
2. A clear ethical reference is needed both to avoid possible unethical practices and to sustain research in the forensic field.
3. Even if moral obligations are different, there is a continuity between the living person and his/her body after death.
4. The Declaration of Helsinki is applicable and useful for forensic research involving human cadavers and remains, promoting the consistency, integrity, and quality of research.
5. Consensus regarding ethical standards and the adoption of national and supranational laws that clearly regulate the topic remains of primary importance for the forensic science community.
